# Occipitocervical Dislocation in Low-Energy Trauma

**DOI:** 10.1155/2018/3931525

**Published:** 2018-11-29

**Authors:** Celeste Tavolaro, Richard Bransford, Aditya Yerrapragada, Carlo Bellabarba, Haitao Zhou

**Affiliations:** Department of Orthopaedics & Sport Medicine, Harborview Medical Center, 325 Ninth Avenue Seattle, WA 98104, USA

## Abstract

Traumatic occipitocervical dislocation (OCD) is described in the literature as a potentially fatal injury secondary to high-energy trauma. We describe a case of OCD occurring in a patient who sustained a ground-level fall whose only clinical symptom was posterior neck pain without neurologic compromise. Computed tomography (CT) and magnetic resonance imaging (MRI) were used to diagnose severe injury to the structurally important ligamentous complex that stabilizes the base of the skull to the spine, along with unstable fractures of the occipital condyle and C1. Emergent surgical instrumentation and fusion of occiput-C2 was performed. Postoperatively, neurologic integrity was maintained. This case illustrates that traumatic OCD is not exclusively secondary to high-energy mechanisms. It also demonstrates that severe neck pain as the only clinical manifestation in a patient with head or neck low-energy trauma is suggestive of a possible OCD. We highlight the importance of the use of head and neck CT as the first imaging-based diagnostic tool to aid in identifying this injury. Finally, surgical stabilization should be performed as soon as possible to minimize neurologic sequelae.

## 1. Introduction

Traumatic occipitocervical dislocation (OCD) is an injury rarely observed [1]. The most common mechanism of OCD is sudden deceleration after high-energy trauma, such as in motor vehicle collisions, pedestrian versus automobile accidents, or falls from great heights. The incidence of OCD is 8% and represents around 20% of the fatal cervical spine injuries [2]. High-energy trauma produces hyperextension of the atlantooccipital joint (AOJ) [3], resulting in severe injury of the osseous and ligamentous complex that stabilizes the skull base to the spine [4]. The considerable force required to cause a traumatic OCD often results in concurrent injuries including the head, spinal cord, or other organ systems [5].

We will describe the first case of traumatic OCD with associated unstable fractures of C0 and C1 in the setting of low-energy trauma. Our purpose is to demonstrate a different traumatic mechanism that can cause this potentially fatal injury, and show that it is not exclusively an injury of high-energy trauma, to prevent delays in diagnosis and treatment to minimize possible neurologic sequelae.

## 2. Case Presentation

**Figure 1:**
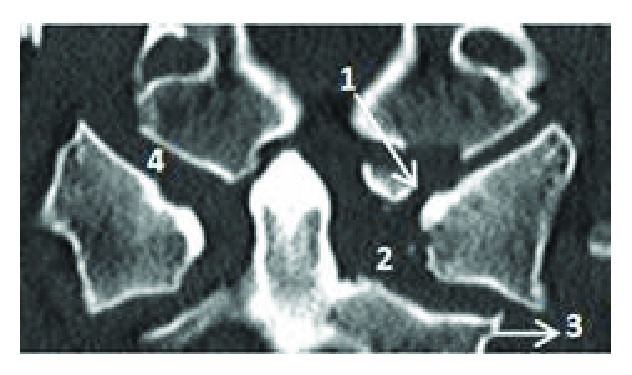
Cervical spine CT scan showing type III left occipital condyle fracture (1), asymmetric left odontoid-lateral mass widening (2), lateral subluxation of bilateral C1 lateral masses (3), and widening of the right atlantooccipital joint (4).

**Figure 2:**
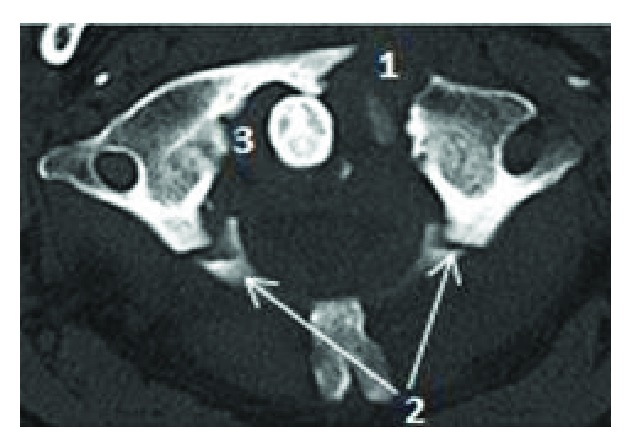
Cervical spine CT scan showing type III Jefferson fracture with anterior arch fracture (1) and posterior arch fractures (2). Note the widening of the atlantodens interval (3).

**Figure 3:**
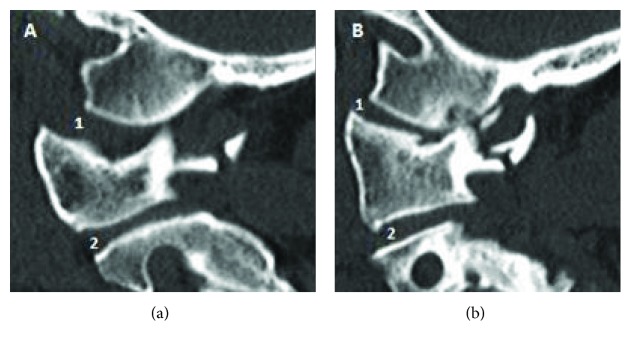
Cervical spine CT scan. (a) Widening and slight anterior subluxation of the right atlantooccipital joint (1) with preserved anatomy of C1–C2 lateral mass joint (2). (b) Slight anterior subluxation of the left atlantooccipital joint (1) and widening of C1–C2 lateral mass joint (2).

A 60-year-old man with a past surgical history of C5–C7 anterior arthrodesis for cervical fracture 17 years ago was found on the floor after an unwitnessed ground-level fall, resulting in a head strike. He was taken to the local hospital in an Aspen collar with a GCS of 15 and no neurologic deficits. Routine ECG and laboratory evaluation was unremarkable except for elevated serum alcohol level. Full cervical spine computed tomography (CT) scan was performed which showed an Anderson and Montesano classification (20) type III left occipital condyle fracture (Figure 1), a Levine and Edwards classification (21) type III (Jefferson) fracture with mild lateral subluxation of bilateral C1 masses (Figure 2), and an asymmetric widening and slight anterior subluxation of the right AOJ (Figure 3). A head CT was performed which showed no acute intracranial abnormalities.

The patient was immediately transferred to a level I trauma center for further management. In the emergency department, he reported severe midline neck pain, worse with movement, and unchanged paresthesia to bilateral upper extremities which he states is his baseline. Physical exam was unremarkable except for a right forehead hematoma with overlying abrasions and tenderness to palpation over the midline posterior neck. He exhibited full strength and unchanged baseline sensation to all extremities. He denied bowel or bladder incontinence and had strong rectal tone and intact perianal sensation. Through his clinical course, he became increasingly altered and agitated, refractory to medication. He was unable to maintain spinal precautions due to his agitation, and in an attempt to protect his cervical spine, he underwent endotracheal intubation.

**Figure 4:**
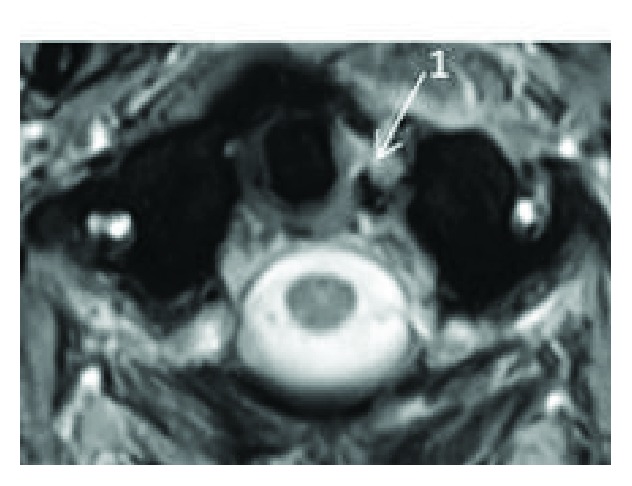
Cervical MRI axial view showing complete disruption of the left transverse ligament (1).

**Figure 5:**
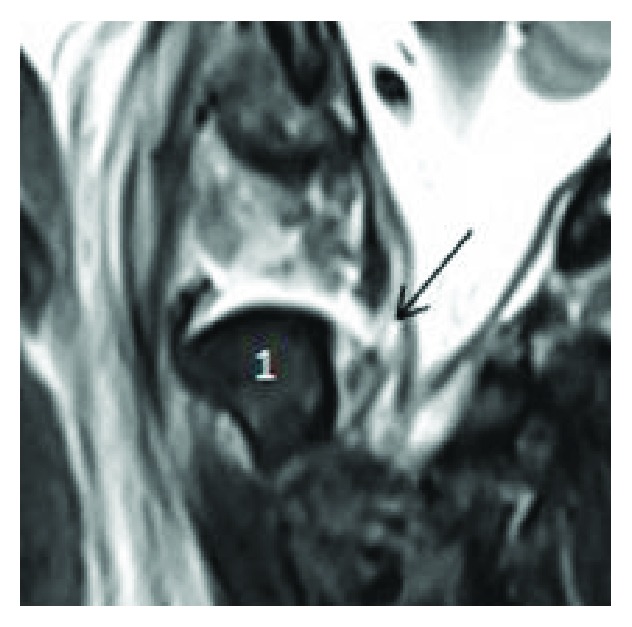
Cervical MRI sagittal view showing the tectorial membrane displaced off the dens (1) and a focal disruption of the left aspect (arrow). Note the prevertebral edema.

**Figure 6:**
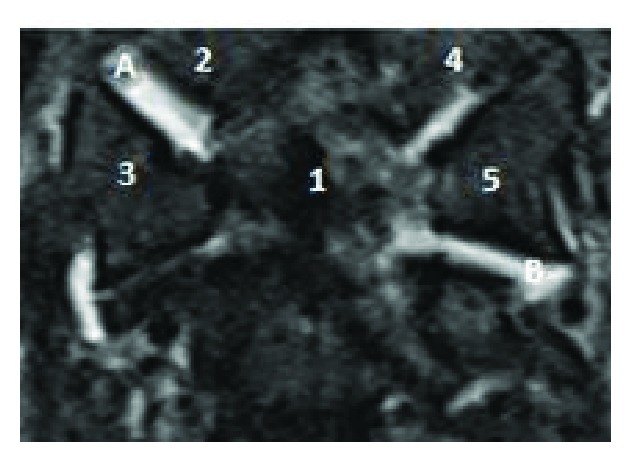
Cervical MRI coronal view. Dens (1), right occipital condyle (2), right C1 lateral mass (3), left occipital condyle, left C1 lateral mass. Note the bilateral edema in the right atlantooccipital joints (A) and the edema in the C1–C2 reflect mild capsular disruption.

**Figure 7:**
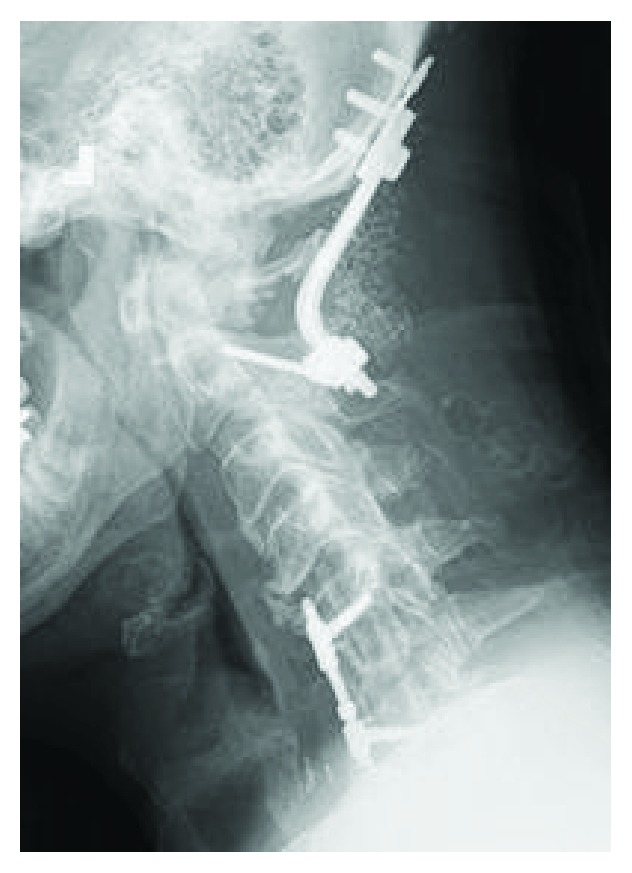
Postoperative lateral radiograph: occiput-C2 fixation with bilateral C2 pars screws and occiput plate connected with precontoured occiput cervical rods. Past surgical history of C5–C7 anterior arthrodesis.

MRI of the cervical spine was performed and showed ligamentous injury at the craniocervical junction (CCJ) (Figures 4 and 5), asymmetric left odontoid-lateral mass widening, widening and subluxation of the right AOJ (Figure 6), and edema of the paravertebral soft tissues around the fracture. CT and MRI established the diagnosis of OCD in a neurologically intact patient. Due to the unstable nature of the fracture, the patient was taken to the operating room for emergent occiput-C2 posterior instrumentation and fusion (Figure 7).

An Aspen collar was in place at all times until surgical stabilization. Immediately, after removing the collar, Mayfield tongs were applied. Prior to patient positioning, baseline somatosensory evoked potentials (SSEPs) were obtained. Using a Jackson table turning frame, the patient was rotated into prone position. Fluoroscopic imaging was used during manual manipulation of the Mayfield apparatus to ensure no further displacement of the fracture.

Occiput to C2 posterior instrumented arthrodesis was performed. Neuromonitoring remained stable during the procedure. The patient was discharged to home five days after presentation.

## 3. Discussion

Due to the small number of published survivors who sustain a traumatic OCD, it is difficult to draw conclusions regarding the mechanisms, symptoms, imaging findings, and treatment strategies for this severe injury [1]. The clinical presentation of patients with traumatic OCD is extremely variable. However, we can divide patients into two broad groups: patients with severe neurological deficits and associated head, spinal cord, or multisystem trauma [4] and patients without neurological deficits who present with only severe neck pain, which represents up to 20% of those with an OCD [2, 6–9]. Our patient above belongs to the second group; the low-energy mechanism and the characteristically wide vertebral canal in the high cervical spine likely contributed to his lack of neurologic deficits.

OCD is described in the literature as a diagnosis often easily missed. Reasons for this include low clinical suspicion, the presence of severe polytrauma, and inexperience with radiographic evaluation of the craniocervical junction (CCJ) [10]. It is also well known that missed diagnoses of OCD have been associated with poor neurologic outcomes and rapid neurologic deterioration [11]. Conventional lateral cervical radiographs that show the variety of radiographic measurements that aid in the diagnosis of OCD may not be sufficient due to the complex osseoligamentous anatomy that cannot be seen on radiographs and the variability in radiographic projections based on the variability of patient anatomy [12–14]. Emergent spine CT as part of the initial advanced trauma life support (ATLS) evaluation has been shown to be much more reliable than standard radiographs to evaluate fractures and joint displacement [15, 16]. MRI may also be necessary to evaluate integrity of the soft tissues, spinal cord, or brainstem [4, 17]. MRI is especially critical in determining the integrity of the major ligamentous structures of craniocervical junction including the tectorial membrane, occipitocervical joint capsules, alar ligaments, and transverse ligament [7, 18]. In addition, dynamic traction fluoroscopy has been proposed as an accurate diagnostic tool for the identification of OCD and as a tool that can help to understand the extent of instability at the CCJ (Table 1) [19].

In our patient, diagnostic CT scan of the cervical spine was performed prior to transfer to our hospital. The CT showed unstable fractures with significant displacement in the upper cervical spine diagnostic of OCD: Anderson and Montesano classification type III left occipital condyle fracture [20], Levine and Edwards classification type III C1 Jefferson fracture [21] with mild lateral subluxation of bilateral C1 lateral masses, and asymmetric widening and slight anterior subluxation of the right AOJ. MRI was performed to evaluate the soft tissues, which demonstrated multiple injuries of the main stabilizing ligaments, including complete disruption of the left alar and left transverse ligament, and focal disruption of the left side of the tectorial membrane. Edema was present in the bilateral facet joints at the AOJ, C1–C2, and C2–C3, reflecting mild capsular disruption. Edema was also noted in the prevertebral and interspinous ligaments spanning C1–C2 and C2–C3.

The literature describes traumatic OCD as a rare injury associated with patients sustaining high-energy trauma, such as motor vehicle collisions, pedestrian versus car accidents, or falls from great heights [6]. The mechanism of traumatic OCD is poorly defined but is always associated with sudden, high-energy deceleration forces resulting in hyperextension, hyperflexion, translation, and/or rotation of the upper cervical spine causing the ligamentous disruption [3, 5, 22–25]. Our case is unique in that it represents the first published case of a patient who sustained an OCD after low-energy trauma.

OCDs are treated with provisional external cervical stabilization in the acute setting followed by urgent surgical stabilization. The ideal form of provisional stabilization is controversial and depends on the timing of OR availability, the degree of initial displacement, the patient's neurologic status, body habitus, and other associated injuries [4]. The definitive treatment should be performed as soon as possible to reduce and stabilize the injured segment to prevent further neurological injury [26, 27]. Craniocervical fixation is the treatment of choice in cases of traumatic OCD, which can be done using a variety of techniques [28]; all of which should involve fusion of the occiput to C2 [7]. In our case, emergent occiput-C2 fixation was performed with bilateral C2 pars interarticularis screws and an occiput plate with three midline screws placed in the occiput keel that were connected with precontoured occiput cervical rods. C1 lateral mass screws were attempted; however, there was too much instability at that level for placement of screws. Neuromonitoring should be performed throughout the operation and in our case, the patient maintained his neurological status throughout.

## 4. Conclusion

Traumatic OCD is not an injury exclusive to high-energy trauma. In some settings, low-energy mechanisms such as ground-level falls with severe neck pain as the only clinical symptom can be suggestive of a possible OCD. Additionally, advanced imaging such as head and neck CT should be used as the first diagnostic imaging modality to aid in identifying this injury. Finally, occipitocervical surgical stabilization should be performed as soon as possible, to minimize neurologic sequelae.

## Figures and Tables

**Table 1:** Harborview classification for craniocervical injuries [10].

Stage	Description of injury
1	Magnetic resonance imaging (MRI) evidence of injury to craniocervical osseoligamentous stabilizers; craniocervical alignment within 2 mm of normal; distraction of ≤2 mm on provocative traction radiography
2	MRI evidence of injury to craniocervical osseoligamentous stabilizers; craniocervical alignment within 2 mm of normal; distraction of >2 mm on provocative traction radiography
3	Craniocervical malalignment of >2 mm on static radiography
